# Pediatric Anesthesiology Special Issue

**DOI:** 10.3390/children8030201

**Published:** 2021-03-07

**Authors:** Camila Walters

**Affiliations:** Division of Pediatric Anesthesia, Vanderbilt University Medical Center, Nashville, TN 37232, USA; camila.walters@vumc.org

## 1. Introduction

Anesthesiology is a medical specialty that provides perioperative care for patients undergoing medical interventions requiring sedation or critical monitoring including surgery, imaging, and other diagnostic and therapeutic procedures. The field was originally a subspecialty of surgery with limited understanding for special considerations of different populations, but now stands on its own with multiple subspecialties. All anesthesia residents are trained in critical care, cardiovascular anesthesia, obstetric anesthesia, acute and chronic pain management, and regional nerve blocks for all age groups. Because anesthesiologists interact with most other medical specialties, anesthesia physicians must show collaboration, communication, and leadership skills to be effective.

In the United States, 6 million children and 1.5 million infants undergo surgery per year [1]. Pediatric anesthesiology focuses on children and the pediatric anesthesia fellowship is especially designed to train physicians on children less than two years of age, with an emphasis on higher acuity pediatric patients that require sophisticated care. The first pediatric anesthetic was realized in 1842, when physicians quickly learned that children have different anesthetic complication profiles than adults [2]. The specialty accounts for the unique clinical and social dynamics of pediatric patients. The current spectrum of specialty clinical and academic activities is broad and includes sedation, critical care, pain management, and perioperative optimization. Recent innovations in pediatric anesthesia include point-of-care ultrasound, regional nerve blocks, and large-scale multi-institution safety and quality initiatives.

The Pediatric Anesthesia Special Issue of *Children* aimed to compile manuscripts written by physician scientists that demonstrate the wide scope of academic practice and interest of pediatric anesthesiologists. The following summarizes the manuscripts presented in the special issue.

## 2. Pediatric Anesthesia Safety

Patient safety efforts have decreased the mortality rate in anesthesiology from 1:2500 to 1:13,000 in 50 years [3,4]. Pediatric anesthesiology has made significant efforts to increase the safety of children undergoing procedures. The subspecialty now has a dedicated journal called Pediatric Anesthesia, and a growing number of national societies, increasing from 13 in 2012 to 20 in 2020 [5]. These societies have ongoing education projects, such as the Safer Anaesthesia from Education (SAFE) pediatric anesthesia course [6].

Pediatric perioperative safety events include major categories such as airway, cardiovascular, and medication errors [7]. Multi-institution collaborations such as Wake Up Safe, Pediatric Airway Registry, and the Pediatric Difficult Intubation Registry, provide data for analysis of events and quality improvement [5,7]. Other large-scale collaborations, including the Pediatric Regional Anesthesia Network and the Pediatric Craniofacial Collaborative Group, allow the compilation of patients undergoing similar procedures to study current practices and improve outcomes in these groups. Large research efforts such as General Anesthesia Spinal (GAS) allow for answers to big-picture questions that address rare issues, such as postoperative cognitive dysfunction, that are difficult to answer within one institution [8]. Studies of specific risk factors for cardiovascular events have led to recommendations, such as consideration of retaining specially trained pediatric cardiac anesthesiologists for complex congenital heart disease patients [7].

One important safety initiative involves the hand-off process. The hand-off process between intensive care unit and perioperative care requires exchange of patient information and is a critical time where missed information may lead to decreased patient safety. Dalal et al. designed a standardized process for pediatric hand-off and found no difference in the time it took to sign out with a significant decrease in the number of missed items post implementation [9].

## 3. Pediatric Anesthesia Specific Considerations

### 3.1. Osteogenesis Imperfecta 

Historically, anesthesiologists have avoided non-invasive blood pressure cuffs (NIBP) or extremity tourniquets in children with osteogenesis imperfecta (OI) to prevent perioperative fractures. A retrospective study of 49 patients who underwent 273 procedures with 90.1% including NIBP cuffs and 22.4% tourniquets found no iatrogenic fractures, indicating that this may be selectively used in OI [10].

### 3.2. Robotic Stereotactic Assistance

Robotic Stereotactic Assistance (ROSA) for Pediatric Epilepsy is a relatively new minimally invasive approach to pediatric epilepsy neurosurgery that has been rarely described in the pediatric anesthesia literature. Patients who had stereoelectroencephalography (SEEG) leads with ROSA in a single-center institution were retrospectively evaluated and found to have no perioperative complications. This technology may improve outcomes in this patient population by avoiding craniotomy [11].

### 3.3. Dexmedetomidine as an Opioid-Sparing Agent in Pediatric Craniofacial Surgery

Pediatric craniofacial reconstruction generally has a high analgesic requirement. A retrospective study of all craniofacial reconstruction surgery patients in one institution did not find a decreased postoperative opioid requirement in patients who received dexmedetomidine versus those who did not receive it, but did find that patients who received higher doses of dexmedetomidine intraoperatively required lower rescue medication for nausea and vomiting postoperatively. Dexmedetomidine may be useful in decreasing these side effects [12].

### 3.4. Perioperative Point-of-Care Ultrasound

Perioperative Point-of-Care Ultrasound (POCUS) has traditionally been used for nerve blocks and line placements and was more recently adopted by anesthesiology for helpful point-of-care diagnostic applications, such as lung ultrasound evaluating for endotracheal position and lung abnormalities, cardiac ultrasound looking for function and volume status, and gastric ultrasound for aspiration risk. This is an innovation in pediatric anesthesia that is being taught and implemented [13].

### 3.5. Anesthetic Implications of Behavior and Emotional Disorders in Children

In addition to common pediatric anxiety and fear of hospitals, children may have behavior and emotional disorders that are challenging in the perioperative setting and require specific management techniques. These techniques may include preparation, medication, Child Life specialists, parental presence, and technology such as smart devices. Tailored approaches may help alleviate patient and family perioperative concerns [14].

### 3.6. Emergence Agitation

Emergence agitation and delirium are common pediatric anesthesia complications. A prospective observational study found a single dose of propofol at the end of a sevoflurane-based myringotomy may decrease postoperative agitation [15].

### 3.7. Preparation of Patients and Families before Pediatric Surgery

A team at Alberta Children’s Hospital surveyed families on how prepared they felt for their child’s surgery. Participants felt more prepared if they completed an in-person program but felt online resources and handouts would be helpful. Based on this information, an online program and video tour were developed to best prepare these families [16].

### 3.8. Hypoglossal Nerve Stimulator for Pediatric Trisomy 21 Patients with Obstructive Sleep Apnea

While hypoglossal nerve stimulators are popular in the adult population, their use is limited in pediatrics. In a case series of trisomy 21 patients with sleep apnea, Karlik et al found a decrease of 97.4% in the apnea-hypopnea index of these patients with stimulator use [17].

## 4. Health Care Inequities

Adult studies have addressed health care inequities based on ethnicity, race, and socioeconomic factors, but there are few inequity studies focused on children in the perioperative period. Lo et al. found that Latino and Black children were less likely to receive a caudal regional block than other racial sub-groups for urologic surgery [18]. Dixit et al. found that although low English proficiency patients received the same amount of opioid medication intraoperatively, these patients had double the odds of receiving opioids in the recovery unit and received higher oral morphine equivalents than English proficient patients [19]. These studies indicate that language and racial disparities may exist in perioperative pain management.

## 5. Conclusions

Having dedicated physicians for the care of children undergoing anesthesia played a vital role in increasing the safety and quality of anesthetic care for the youngest and most vulnerable patients. The Pediatric Anesthesia Special Issue of *Children* provided a bird’s-eye view of the broad scope of pediatric anesthesia, including collaboration, research, education, and quality improvement for the many facets of pediatric perioperative care.
